# Effects on the Hypothalamo-Pituitary Axis in Patients with CNS or Head and Neck Tumors following Radiotherapy

**DOI:** 10.3390/cancers15153820

**Published:** 2023-07-27

**Authors:** Jordan Bouter, Yves Reznik, Juliette Thariat

**Affiliations:** 1 Radiotherapy Department, Centre François Baclesse, Avenue du Général Harris, 14000 Caen, France; j.bouter@baclesse.unicancer.fr; 2 Department of Endocrinology, University Hospital of Caen, Avenue de la Côte de Nacre, 14033 Caen, France; reznik-y@chu-caen.fr; 3 Corpuscular Physics Laboratory, ENSICAEN, Boulevard Maréchal Juin, 14050 Caen, France; 4 Unicaen—Normandie Université, 14050 Caen, France

**Keywords:** tumor, brain, head and neck, hypopituitarism, endocrine, radiotherapy

## Abstract

**Simple Summary:**

The effects of radiation on the hypothalamic-pituitary axis have been discussed for several decades, but many unknowns remain. Among them, the hypothalamic and/or pituitary dose at risk of causing a deficit, the rate and time of onset of the latter and the possible superiority of one irradiation technique over another. This information will allow a more appropriate follow-up of each patient and early management of radiation-induced toxicities. Reported neuroendocrine impairment after radiotherapy is very heterogeneous. We found new deficiencies occurred in 40% of patients within a median follow-up of 5.6 years. We found differences in sensitivity between the anterior pituitary axes, with the growth hormone axis being the most easily damaged by irradiation and the thyroid hormone axis the least sensitive. Pituitary gland protection and early detection of deficiencies need further investigations.

**Abstract:**

Background: Knowledge about the precise effects of radiotherapy on hypothalamo-pituitary functions is limited. Reduction of side effects is a major goal of advanced radiotherapy modalities. We assessed strategies for monitoring and replacement of hormone deficiencies in irradiated patients. Methods: A search strategy was systematically conducted on PubMed^®^. Additional articles were retrieved to describe endocrine mechanisms. Results: 45 studies were evaluated from 2000 to 2022. They were predominantly retrospective and highly heterogeneous concerning patient numbers, tumor types, radiotherapy technique and follow-up. Endocrine deficiencies occurred in about 40% of patients within a median follow-up of 5.6 years without a clear difference between radiotherapy modalities. Somatotropic and thyrotropic axes were, respectively, the most and least radiosensitive. Conclusions: Current pituitary gland dose constraints may underestimate radiation-induced endocrine deficiencies, thus impairing quality of life. Little difference might be expected between radiation techniques for PG tumors. For non-PG tumors, dose constraints should be applied more systematically.

## 1. Introduction

Patients with brain and skull base/head and neck tumors may experience pituitary gland (PG) deficiencies caused by tumor-specific or treatment-related mechanisms. Delayed detection of such hormone deficiencies may participate to increased morbi-mortality. Hypopituitarism has been linked to premature mortality [1], predominantly from cardiovascular and respiratory disease, with risk factors identified such as age at diagnosis, female gender, and craniopharyngioma-type lesions [2]. Hypopituitarism is associated with substantial morbidity, such as vertebral fractures [3]. 

Radiotherapy (RT) aims at accurately delivering an effective radiation dose to the target tumor volume while limiting the dose received by surrounding normal tissues to minimize the risk of long-term toxicities. 

Irradiation techniques have greatly evolved over the past few decades. The choice of technique for PG or non-PG tumors depends on several factors. 

3D conformal radiation therapy (3DCRT) (including whole brain RT (WBRT), either normofractionated or moderately hypofractionated, and total body irradiation) use photons; they have been widely available for years but leave little possibility of OAR sparing. Intensity-modulated radiotherapy (IMRT) has been implemented since the 2000s and is now widely implemented. IMRT relies on several beams (typically 5–9 or 1–2 beam arcs), each highly modulated with photon fluence to deliver its highly conformal irradiation. IMRT allows better sparing of healthy tissues than 3DCRT from high doses at the cost of larger volumes receiving low doses, and is usually normofractionated, i.e., uses 1.8–2 Gy fractions. Stereotactic radiosurgery (SRS) and hypofractionated (i.e., using high doses per fraction) stereotactic body radiotherapy (SBRT) using photons deliver multiple beams (typically 50–200) stereotactically. SRS and SBRT deliver high-dose gradients, thus achieving good organ at-risk (OAR) sparing. Yet, the larger the tumor, the larger the high “red shell” dose in surrounding tissues. Therefore, normal tissues sensitive to hypofractionation due to their low radiobiological α/β ratio theoretically limit the use of SRS/SBRT to small tumors, and caution should be exerted for OAR in close proximity to the tumor. The limiting tumor size is not consensual to date: while a maximal diameter of 3 cm may be proposed for intracranial tumors, this size threshold is dependent on OAR proximity and can be compensated for by decreasing the dose per fraction (down to normofractionated schemes using 1.8–2 Gy fractions, i.e., normofractionated SBRT, which is less frequent nowadays). Of note, the dose equivalence calculation model between various fractionation schemes is invalid for doses per fraction greater than 6–8 Gy, as can be the case for SRS/SBRT. 

Proton therapy is further indicated to spare healthy tissues from low doses due to its physical properties. Proton therapy gradients may not systematically be better than with IMRT or SRS/SBRT when the proximity between OAR and tumor is in the order of a few mm. Access to proton therapy remains limited, leading to low recruitment and limited publication numbers. 

The prerequisite for preserving the PG in the context of brain/skull base head and neck tumors, like any other organ, is that it is delineated on the RT planning computed tomography (CT). Without delineation, the dose to the organ can only be roughly estimated visually but can neither be minimized during the optimization process nor calculated. Accurate delineation may require CT and MR imaging co-registration to define tumour and organs better. 

Dose constraints are mostly defined from retrospective data [4] to accept a maximal 5%-risk of severe toxicities. This clinical trade-off illustrates a trade-off between tumor control and toxicities as no radiotherapy modality can give 100% dose to the tumor and 0% to all organs at risk. 

The description of radiation-induced pituitary deficiencies began in the 1990s [5]. The pituitary gland is a small pear-like 1 cm^3^ organ made of anterior/posterior lobes situated in a complex anatomic area nearby the brainstem and chiasma, which are top priorities along with the tumor during RT planning optimization. Historically, a soft objective, such as the ALARA (As Low as Reasonably Achievable) dose principle, was applied to the PG with poorly modulated radiotherapy. With modern techniques, when dose constraints to the hypothalamo-pituitary axis cannot be met without compromising treatment efficacy, the risk of hypopituitarism is very high. Because hormone replacement therapies are available, the challenge is early detection of hypopituitarism and estimation of the risk/benefit of replacement therapies.

Yet, monitoring pituitary deficiencies is not systematic after irradiation therapy of the skull base, and side effects are likely underestimated. Cellular components of the different hormonal pituitary axis are intermingled within the anterior pituitary lobe and are variably radiosensitive owing to dose dependency and time delay until hormone deficiency appearance [6]. There are currently no guidelines describing how the pituitary axis should be monitored and how the patient’s age, tumor site, tumor proximity to the pituitary/hypothalamus may influence radiotherapy damage. Systematic data collection on correlations between the dose administered to the pituitary gland and the appearance of hormone deficiencies is warranted to define dose constraints for radiotherapy optimization and to allow early detection of such hormone deficiencies, which might prevent consequent morbidity. 

The complex identification of endocrine deficiencies occurring following irradiation is generally devoted to endocrinologists. However, understanding radiation-induced damage to the hypothalamo-pituitary axis and their time appearance is critical for radiation oncologists correlating radiation parameters, i.e., total dose, fractionation and dose per fraction, dose distribution/heterogeneity, and pituitary damages. This is also a prerequisite to defining relevant dose constraints to the pituitary gland and hypothalamus during the radiotherapy planning optimization step. 

Our review focuses on the impact of pituitary irradiation on the hypothalamo-pituitary axis to provide keys to oncologists for hormone testing, dose constraints, follow-up, and hormonal management of adult patients undergoing radiotherapy for brain or head and neck tumors. Hypopituitarism following radiotherapy during childhood is out of the scope of the present review.

## 2. Materials and Methods

A search strategy was systematically conducted on the Pubmed^®^ database with the following MESH terms (“Radiotherapy/adverse effects”[Mesh]) AND ((“Pituitary Gland/radiation effects”[Mesh]) OR (“Pituitary Hormones, Anterior/adverse effects”[Mesh] OR “Pituitary Hormones, Anterior/radiation effects”[Mesh]) OR “Hypopituitarism/etiology”[Mesh]) and the following keywords {“cranial radiotherapy” OR “cranial irradiation”} AND {“hypopituitarism” OR “pituitary deficiency”}. Articles ranging from 2000 to 2022 and reference lists were evaluated for other literature. Additional articles were retrieved to describe endocrine mechanisms and current testing, follow-up, and therapeutic strategies. 

## 3. Results

**Figure 1:**
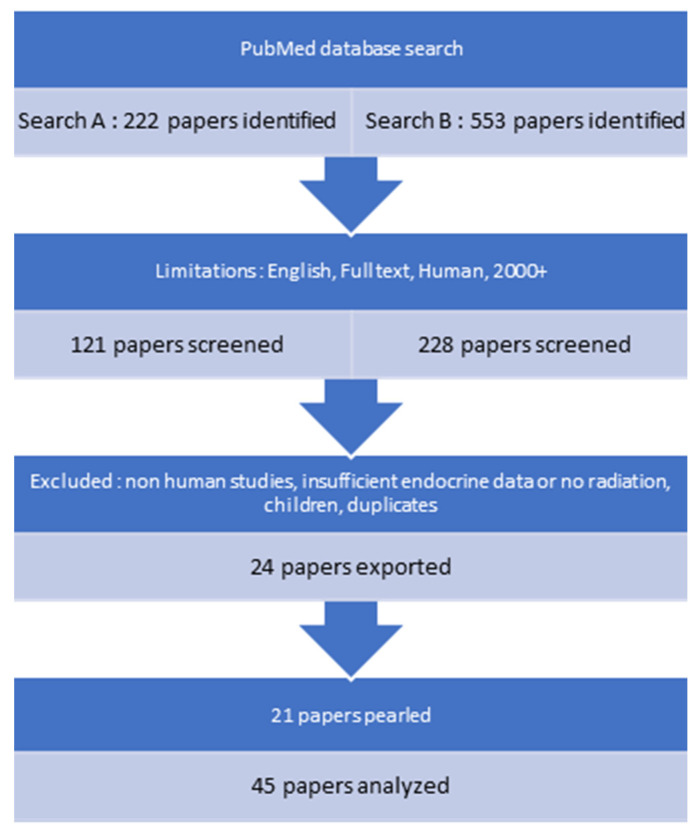
PRISMA search.

The current review investigated PG deficiencies. Forty-five articles fulfilled our search criteria (Figure 1). 

Forty-five articles were analyzed (Table 1 and Table 2); 89% (40/45) were retrospective, with their inherent biases due to clinical data collection constraints and missing data, and they were often small (<100 patients). There was significant heterogeneity in tumor types, radiotherapy techniques, pituitary dose and follow-up.

### 3.1. Tumor Types

Median number of patients per study was 61, ranging from 20 to 656 (Table 1 and Table 2). The median patient number was 52 (20–436) in non-PG studies and 78 (27–656) in pituitary adenoma studies. Overall, pituitary adenomas were overrepresented (N = 24 studies), followed by various central nervous system (CNS) tumors including brain metastases undergoing prophylactic or therapeutic whole brain irradiation (N = 6), meningiomas (N = 4), gliomas (N = 4), nasopharyngeal tumors (N = 3), chordomas/chondrosarcomas (N = 2), sinonasal tumors (N = 2), or hematological tumors undergoing total body irradiation (N = 2). 

In non-PG studies, the mean age was 46.6, ranging from 13 to 90 (Table 1). The mean follow-up was 6.2 (±3.7) years, ranging from 0.25 to 35 years. In pituitary adenoma studies, the mean age was 48.7 years, ranging from 6 to 93 years, and the mean follow-up was 5.8 (±2.5) years ranging from 0.5 to 23.2 years.

### 3.2. Radiotherapy Modalities

1. Pituitary radiotherapy modality in non-pituitary tumors
  According to our literature search, non-PG tumor series (21 studies) were treated with 3DCRT in 12, IMRT in one, SBRT in one, and protons alone or combined with photons in four studies. Thus, normofractionated photon and proton techniques appeared as the most commonly used for non-PG tumours.
2. Pituitary radiotherapy modality in pituitary tumors
  In contrast, 88% (21/24) of pituitary adenoma studies used SRS (single fraction) (16/24) or SBRT (6/24) in 3 to 5 fractions. The remaining three studies used 3DCRT. Thus, SRS/hypofractionated SBRT appeared as the most commonly used radiotherapy technique for PG tumours.
3. Pituitary doses in non-pituitary tumors
  In non-PG, the mean dose to the pituitary gland was 47.6 (±12.9) Gy, ranging from 0 to 79 Gy (calculated from historical data of series indicated in Table 1). Most studies used photons (13/23), almost exclusively 3DCRT and the mean dose delivered to the pituitary gland was 47.2 (±9.4) Gy, ranging from 6 to 73 Gy. Four studies [10,11,15,27] used protons alone or associated with photons to treat gliomas, meningiomas, chordomas or chondrosarcomas. Among these studies, the mean pituitary dose was 57.9 (±9.2) Gy, ranging from 0.6 to 72.8 Gy. Six studies did not report the radiation technique, and five did not report the dose to the pituitary gland.
  Different total doses to the PG seemed to result in different reported pituitary deficiency rates, as illustrated by a 14% rate of patients developing new pituitary impairments after a median dose to the pituitary gland of 13 Gy and a median follow-up of 16 years [16] opposed to a 93% rate after 46 Gy and a median follow-up of 2 years [17]. However, such variations leave little confidence in interpreting deficits as they originate from retrospective studies. Prospective studies with systematic dosages are still needed, assuming that the excess cost of endocrine monitoring would probably be compensated by reducing the health expenses associated with managing complications.
4. Pituitary doses in pituitary tumors
  Considering pituitary adenomas, the mean dose to the PG across studies was 19.7 (±4.2) Gy for SRS, 34.7 (±14.4) Gy for SBRT (52.2 for normofractionated SBRT and 26.0 for hypofractionated SBRT) and 49.1 (±3.7) Gy for 3DCRT. The mean tumor volume treated by SRS was 3.1 ± 1.6 cm^3^ and 4.4 (±0.7) cm^3^ by hypofractionated SBRT (calculated from historical series in Table 2). Only one article reported tumor volume treated by normofractionated SBRT and another by 3DCRT, respectively 8 cm^3^ and 1.9 cm^3^ [35,40]. As pituitary dose was not reported in SRS studies, Table 2 lists the margin dose, i.e., the isodose including the tumor, here the PG.

### 3.3. Hypothalamus Dose

In addition to the pituitary gland, radiation dose to the hypothalamus may be a risk factor for hypothalamo-pituitary dysfunction. However, this information is rarely reported. Four non-PG tumors series reported mean hypothalamus dose for meningiomas [7], nasopharyngeal tumors [17] and sinonasal tumors [22,23]. Mean hypothalamus doses were 15.8 Gy, 10.5 Gy, 48.0 Gy and 39.5 Gy, respectively. Higher hypothalamus dose was not linked to higher pituitary deficiency rates. Two pituitary adenoma series with SRS also reported hypothalamus dose [31,42]. Mean hypothalamus doses were 1.7 Gy and 1.0 Gy, respectively 30% and 43.5% of new pituitary deficiencies. Further studies on neuroendocrine impairment after radiation should report pituitary and hypothalamic doses.

### 3.4. Pituitary Hormone Deficiencies by Endocrine Axis

As previously shown, pituitary deficiencies have different outcomes according to the damaged axis. More frequent deficits or deficits occurring after lower doses could reflect differential sensitivities across endocrine axes. Improvements during follow-up after irradiation have been achieved by targeted monitoring. Yet, several parameters interfere, and published studies show great heterogeneity. This heterogeneity is partly due to the different diagnostic criteria for pituitary deficits, as some studies did not use dynamic tests, and others only reported supplemented deficits.

5 Pituitary hormone deficiencies in non-pituitary tumors

Considering non-pituitary tumors, overall endocrine deficiencies occurred in 51.5 (±26)% for any axis, 22.8 (±17.5)% for prolactin, 20 (±16.2)% for ACTH, 16.2 (±12.8)% for TSH, 20.7 (±11.2)% for FSH/LH and 33.7 (±29.1)% for GH (Table 1). In some studies, endocrine testing was only performed on clinical suspicion [24]. Other studies only performed a single time point endocrine assessment [19,22,24,25]. In one of the prospective studies, the median number of endocrine tests per patient was only 1.0 (range: 1.0–2.0) [20]. Most of these studies do not report the number of endocrine function assessments per patient. These figures possibly reflect the lack of medical or patient adherence to repeat blood test monitoring over long periods, thus potentially underestimating the actual rates. 

Prolactin axis dysfunction is frequent (22.8%) but may be underestimated as one study showed no prolactin dysfunction in 26 patients after a median follow-up of 2.7 years [20], and two studies reported hyperprolactinemia only in women for tumors both close and distant to the HP axis [12,14]. 

Corticotropic deficiency seems to occur in about 20% of patients, but the ACTH deficiency rate was highly variable among studies. Thus, in a study, the ACTH deficiency rate was the same as GH deficiency (>70%) [17] while being very low (4%) in another [26].

Concerning the thyrotropic axis, its deficit is the rarest (16.2%), and this axis has also been reported to be the most radioresistant [12]. It is unclear whether patients get a replacement in case of biological deficiencies. However, symptoms are well recognized and would trigger replacement to avoid deterioration of the quality of life and long-term consequences. 

Gonadal deficiency also concerned around 20% of patients, but the role of hyperprolactinemia and the possible hormonal replacement were exceptionally discussed. 

Somatotropic axis deficiency is the most frequently reported dysfunction in >30% of patients (range 0–90%) in analyzed papers [10,14]. This is consistent with the higher radiosensitivity previously described [12]. However, dynamic tests were not always performed and could have led to higher rates. 

Moreover, HP deficiencies may be isolated or involve multiple axes, up to panhypopituitarism. This information is rarely reported. Some studies have found 0% [8] to 16% [25] single deficiencies with >45 Gy in the pituitary gland. Lamba et al. described HPT deficiency as most frequently associated with at least one other HP deficiency [11]. A study reported no multiple axes deficiencies, but the median, mean dose to the pituitary gland was low (13 Gy) [16]. With a median, mean dose to the pituitary gland of 34.4 Gy, another study found that 2/3 of new pituitary impairments concerned a single axis [9]. These results suggest that the number of new deficient HP axes increases with the mean dose to the pituitary gland. 

**Figure 2:**
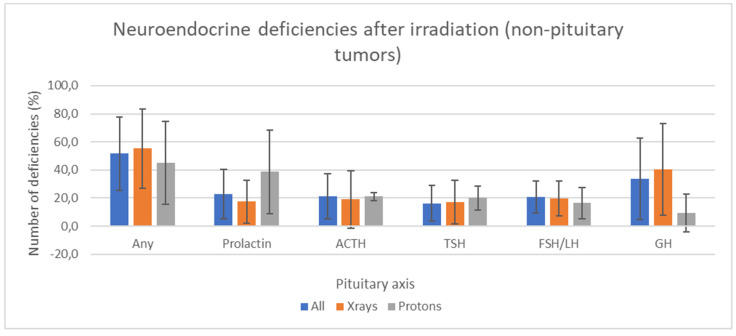
Pituitary axis deficits in non-pituitary tumors depend on the type of radiotherapy. This figure represents the averages and standard deviations of the mean deficiency rates between the studies without directly comparing X-rays and protons. The overall mean dose to the pituitary gland was 47.6 (±12.9) Gy. The mean dose delivered to the pituitary gland was 47.2 (±9.4) Gy with photons and 57.9 (± 9.2) Gy in articles which used protons. Regardless of the particle, pituitary deficiencies were 51.5 (±26)%, 20.1 (±18.2)%, 20 (±16.2)%, 16.2 (±12.8)%, 20.7 (±11.2)% and 33.7 (±29.1)% for respectively any axis, prolactin, ACTH, TSH, FSH/LH and GH.

Figure 2 reports the pituitary axis deficits after photon or proton radiotherapy. Considering the variability, no difference appeared between photon and proton studies despite a Dmean 10 Gy higher in the latter.

6 Pituitary hormone deficiencies in pituitary tumors

Regarding pituitary adenomas, deficiency of any axis occurred in 27.1 (±14.1)% after SRS, 5.8 (±7.7)% after hypofractionated SBRT (hf SBRT), 36.7% after normofractionated SBRT (nf SBRT) and 88% after 3DCRT. ACTH deficiency occurred respectively in 11.4 (±6)%, 1.7 (±2.4)%, 28.6% and 29.7 (±25.4)%. TSH deficiency was 14.8 (±9)% after SRS, 1.7 (±2.4)% after hf SBRT, 32.3% after nf SBRT and 32.7 (±28.9)% after 3DCRT. 12.8 (±9.5)% new FSH/LH deficiency appeared after SRS, none after hf SBRT (1 article), 13.9% after nf SBRT and 39.7 (±49.2)% after 3DCRT. GH deficiency was not reported after nf SBRT but occurred in 10.9 (±10.4)% after SRS, not after hf SBRT (1 article) and 98% after 3DCRT. Prolactin deficiencies were not reported.

**Figure 3:**
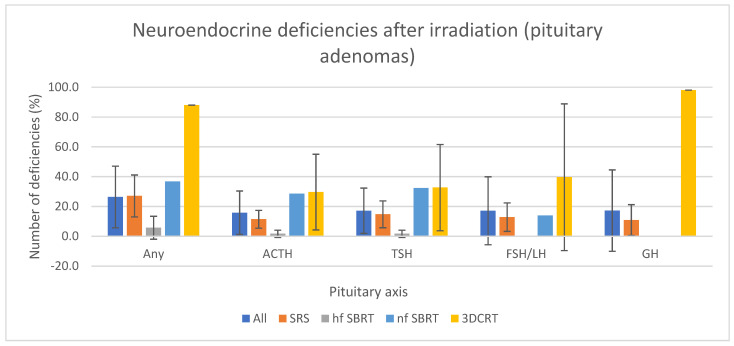
Pituitary axis deficits in non-pituitary tumors depend on the radiotherapy technique. This figure represents the averages and standard deviations of the mean deficiency rates between the studies without directly comparing techniques. The mean dose to pituitary across studies was 19.7 Gy for SRS, 26.0 Gy for hypofractionated SBRT (hf SBRT), 52.2 Gy for normofractionated SBRT (nf SBRT) and 49.1 Gy for 3DCRT. Deficiency of any axis occurred in 27.1 (±14.1)% after SRS, 5.8 (±7.7)% after hf SBRT, 36.7% after nf SBRT and 88% after 3DCRT.

Overall, endocrine deficiencies occurred in 26.4 (±20.7)%, 15.8 (±14.6)%, 17.1 (±15.2)%, 17.1 (±22.8)% and 17.2 (±27.3)%, respectively any pituitary axis, ACTH, TSH, FSH/LH and GH. Figure 3 shows the pituitary axis deficits according to the radiotherapy technique. 

Patients with pituitary adenoma, most of them secretory and previously operated on, experienced an overall lower rate of new pituitary deficiencies than non-pituitary tumor patients. That could be explained by the higher prevalence of HP impairment before radiotherapy. Moreover, interpretation should be cautious, as no controlled comparative studies exist.

Hypofractionated SBRT seems to cause lower pituitary deficiency rates than SRS for similar mean tumor volume. However, about 2/3 of papers reporting pituitary deficiencies after pituitary tumor irradiation used SRS with Gamma Knife^®^ (as opposed to 4/24 for hf SBRT), and deficiency rates were highly variable for a given technique. Therefore the comparison between techniques is difficult. Moreover, high doses per fraction in SRS exceed the validity limits of the common radiobiological models and are, therefore, difficult to compare to less hypofractionated regimens. 

The median time between radiation and diagnosis of any new pituitary dysfunction was rarely reported and ranged from 0.9 years in a prospective study in low-grade glioma patients [10] to 5.6 years in a retrospective study in sinonasal cancer patients [23]. However, for example, an ACTH deficiency may occur as early as two months after the end of radiotherapy, as reported by Lamba et al. [11]. GH deficiency could appear more rapidly the higher the dose received [9]. However, the pituitary gland received a high radiation dose in all eight articles (pituitary adenomas and non-pituitary tumors), reporting the median time between irradiation and GH deficiency diagnosis. 

### 3.5. Posterior Pituitary Gland Deficiency

ADH deficiency after irradiation seems to be rare, but underreporting is also likely. Only one non-pituitary tumor study reported a single case of diabetes insipidus after irradiation [45]. This patient had a pituitary adenoma and underwent at least one surgery before irradiation, but no additional information was given. Two pituitary tumor studies reported 1.1% and 3.5% of new diabetes insipidus after SRS [37,38]. To our knowledge, radiation-induced oxytocin deficiency has never been reported. The posterior pituitary gland may seem radioresistant because, unlike its anterior counterpart, ADH and oxytocin are produced in the hypothalamus and then stored in the posterior pituitary before release [52]. Oxytocin deficiency is probably unassessed because measuring plasma levels remains challenging, and its clinical impact is low. 

### 3.6. Radiotherapy Planning

Few papers have proposed dose thresholds leading to any hypothalamo-pituitary axis deficit. Mean dose to the pituitary gland >45–50 Gy seems to increase the risk of developing a pituitary deficiency [7,8,27,29], but the threshold may be lower (Dmean > 30 Gy) [16] and needs to be refined. Kyriakakis et al. proposed PG Dmean thresholds for each axis: 10 Gy for GH, 30 Gy for FSL/LH, 32 Gy for ACTH and 40.8 Gy for TSH [12]. Considering SRS, an article identified PG Dmean > 10 Gy as a potential risk factor for developing pituitary deficiency [29]. 

A study focused on the use of generalized equivalent uniformed dose (gEUD) as an alternative predictor of endocrine deficiency and reported a dose inducing 50% of complication (TD50) of 60.6 Gy (95%CI 59.1–62.0 Gy) to the pituitary gland for deficiency of any axis [15]. gEUD was initially developed for tumors and was defined as the biologically equivalent dose that, if given uniformly, would lead to the same cell kill in the tumor volume as the actual non-uniform dose distribution. It was then adjusted to also apply to normal tissues [53].

Regarding the hypothalamus, a relatively low mean dose (20 Gy) has been identified as a potential risk factor for developing hypothalamic-pituitary deficiency [7], and further research is needed to define the role of hypothalamic dose better. 

**Figure 4:**
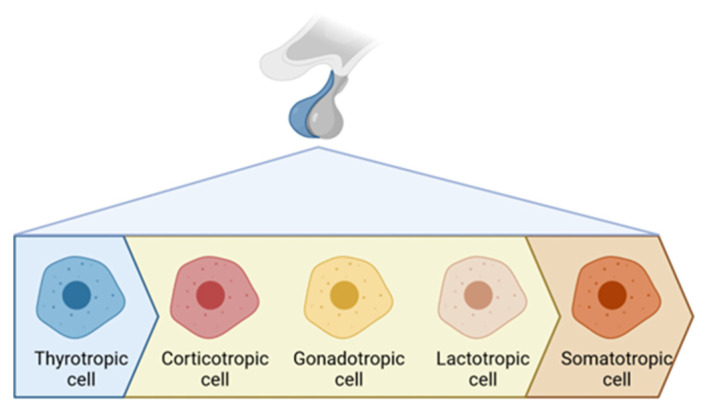
Schematic representation of cellular radiosensitivity gradient in the anterior pituitary gland. The HPS axis demonstrates the highest sensitivity, while the HPT axis shows the lowest. Other axes seem to have an intermediate sensitivity.

Figure 4 proposes a summary of the radiosensitivity gradient within the pituitary gland. However, it appears difficult to propose dose thresholds for each axis from available data. 

## 4. Discussion

As previously stated, the analyzed series showed great heterogeneity in tumor types, radiation technique, the radiation dose to PG and fractionation. PG tumors have lower rates of new deficits than non-PG tumors, likely due to a higher rate of pre-irradiation deficits and, in some series, to higher tumor doses (in the case of malignant tumors) for non-PG tumors. Median follow-up ranged from 1.7 to 16.1 years, while the onset of new pituitary deficiency could occur more than ten years after irradiation, thus underestimating impairment rates. Deficiency rates may also be inaccurately estimated by the different definitions of pituitary impairment as some studies did not use dynamic tests, and some others only reported deficiencies with hormone replacement. Retrospective studies only reported patients with pituitary function data available. Moreover, some studies reported only one pituitary function assessment per patient at various times. Propensity scores and patient registries could provide better-quality data. 

The evaluation of the effects of supplementation is absent from almost all studies, although it could be helpful, particularly for non-pituitary tumors. 

Multiple radiation techniques were used across studies, each with its advantages and limitations, for different tumors whose distance from the pituitary gland was generally unknown. Moreover, techniques have evolved, making it even more difficult to compare them. Another bias is introduced as SRS, SBRT, or proton techniques may not be easily available. Comparative studies are needed to address whether one technique is superior to others in a specific setting. A temptative illustration of the pros and cons of current radiotherapy techniques used for pituitary and on-pituitary tumors close to the pituitary gland is given in Table 3. 

Radiotherapy optimization at the planning phase relies on empirically defined dose thresholds for each OAR in or near the treatment fields. Such dose thresholds are converted into “dose constraints” that, if not fulfilled, require changes in beam arrangements of 3DCRT or any other forward-planning radiotherapy. For modern radiotherapy techniques, such as IMRT, SBRT or pencil beam scanning proton therapy, treatment planning usually relies on specific softwares (treatment planning software, TPS) that include inverse planning optimization algorithms. Dose constraints are set as high-priority parameters if damage to the organ can be life-threatening and cannot be compensated by any medication. Under such circumstances of potential damage to a critical organ, dose thresholds are usually well identified for their correlation with toxicity/risk. The recommended dose constraints are usually calculated to represent the usual acceptability threshold of 5% of severe toxicities. This means that the priority would be made on the critical OAR rather than on the tumor, i.e., the radiation oncologist would accept to reduce the dose to the tumor slightly. This is typically the case for serial organs, such as the spinal cord and brainstem. 

In pituitary tumors, damage to the chiasma would follow the same rule. In contrast, for non-pituitary tumors next to the pituitary gland (here considered an OAR), any damage to the gland might be substituted by adequate endocrine medication, partially explaining the various doses received across studies (Table 1 and Table 2). 

Recommended dose constraints are PG Dmean ≤ 45 Gy [54] and Dmax < 45–50 Gy [4], while the 5-year rate of hypopituitarism with Dmax ≤ 45 Gy to whole pituitary gland has been estimated at 20–40% ([55]). When hypofractionation is used, α/β = 2 Gy should be considered for the PG [54]. 

As stated previously, the hypothalamic dose report lacks standardization. Moreover, there is currently no recommended dose constraint for the hypothalamus in adults. 

Although each axis has its specific features, a deficit in any of them can result in significant morbidity, justifying the need for monitoring and adequate substitution. GH deficiency results in increased risks of metabolic syndrome (1.3–2-fold) and cardiovascular deaths (1.5-fold) [56]. GH deficiency also induces changes in body composition (increased body fat, decreased lean body mass), osteopenia, osteoporosis, impaired glucose metabolism and impaired physical status [57,58]. GH substitution may be considered in adults with severe GH deficiency (defined as a peak GH response of <9 mU/L during a dynamic test) to improve quality of life [56], but cardiovascular and metabolic benefits and mortality are controversial [56,57,59]. In patients over 60, GH therapy showed an improvement of lean mass/fat mass ratio with no effect on strength but an increased risk of adverse effects. Drawbacks of GH therapy include oedema, arthralgia, impaired glucose tolerance and mis-adherence to daily subcutaneous injections [60]. 

Severe prolactin deficiency may indicate extensive pituitary damage [61]. On the other hand, hyperprolactinemia causes gonadotropic deficiency and infertility in both genders. To date, there is no rationale for hormone replacement in prolactin deficiency. Asymptomatic non-prolactinoma-related hyperprolactinemia can be left untreated. However, hyperprolactinemia-induced hypogonadism should be treated [62]. 

In men, hypogonadism manifests as sexual dysfunction, small testes, anemia and loss of secondary sex characteristics such as body hair. Hypogonadism also leads to nonspecific symptoms such as fatigue, sleep disturbance or poor concentration [63]. In young women, hypogonadism results in early premature menopause, with menopause-like consequences such as metabolic disorders and cardiovascular risk increase [64]. In contrast, postmenopausal women with ovarian deficiency may not have increased morbidity [65]. Long-term effects of hypogonadism also include an increased risk of developing metabolic syndrome and type 2 diabetes [66]. It also results in anxiety, depression, low self-esteem and altered body image, which is frequently underestimated by physicians [67]. Estrogen substitution may be cautious in women mainly because of increased breast cancer risk [68]. Women with hypopituitarism are less likely to undergo estrogen substitution than the hormonal substitution of another axis [69]. The Endocrine Society recommends estrogen replacement in premenopausal women but omits recommendations in postmenopausal women [62]. In men, the benefits of testosterone replacement are variable. It may prevent anemia and improve bone mineral density, muscle mass/fat mass ratio and sexual function [62]. 

Hypothyroidism can present non-specific symptoms, including fatigue, weight gain, constipation, dry skin, or chilliness. Hypothyroidism causes (diastolic) hypertension by increasing systemic vascular resistance, endothelial dysfunction, impaired cardiac relaxation and contractility and increased risk of coronary heart disease [70]. Anemia, decreased insulin sensitivity, and reproductive disorders are also reported [71]. Central hypothyroidism usually presents with mild or no (15%) symptoms [72].

L-T4 therapy can improve symptoms and should be introduced after adequate substitution of adrenal deficiency [62]. 

Secondary adrenal insufficiency (AI) may be a life-threatening condition characterized by glucocorticoid deficiency without aldosterone alteration. AI may present as an acute adrenal crisis precipitated by physiological stress with symptoms including vomiting, abdominal pain, myalgia, severe hypotension, and hypovolemic shock [73]. More often, patients have various nonspecific symptoms, such as weight loss, fatigue, myalgia, alabaster, or moderate abdominal pain, and/or syndrome of inappropriate antidiuretic hormone secretion (SIADH)-like euvolemic hyponatremia [74]. Secondary AI alters the physical and mental quality of life [75] and induces a 6–7-fold increase in cardiovascular or infection-related mortality rate [76].

Immediate 50–100 mg hydrocortisone injection is advocated when suspecting acute AI. Hormone replacement by hydrocortisone is recommended at a dose of 10–15 mg daily for secondary adrenal deficiency.

Quality of life with hypopituitarism has been assessed almost exclusively in patients with functioning or non-functioning pituitary adenoma. These patients often have poor quality of life worsened by radiotherapy and hypopituitarism [77]. Moreover, hypopituitarism patients may benefit from hormone replacement, but they generally maintain a worse quality of life than healthy controls [78]. This encourages pituitary gland sparing when achievable regarding tumor control. If not, the dose received by the gland should be reported, and the patients should be monitored for pituitary deficiencies to reduce the quality of life impairment by precociously introducing hormone replacement.

Surgery of the pituitary region frequently leads to hypopituitarism, and the risk is correlated to tumor size and extension in the pituitary gland [79]. Most patients in the reviewed articles underwent surgery before radiation, but no data about pituitary region involvement in non-pituitary tumors was available. 

Chemotherapy is generally not considered a cause of hypopituitarism, although it could potentially sensitize to the effects of radiation [16]. In reviewed papers, patients were rarely exposed to chemotherapy, with the exception of nasopharyngeal cancers. However, a paper on nasopharyngeal tumors reported 82.9% of new hypothalamo-pituitary dysfunction after chemoradiation as opposed to 17% after radiotherapy alone [19], while another found no significant difference in adding chemotherapy [16]. 

## 5. Conclusions

Endocrine deficiencies are frequent following irradiation of the pituitary gland in the context of brain and head, and neck tumors. In addition, current data do not allow highlighting of significant differences between radiotherapy techniques. Current recommendations regarding dose constraints for the pituitary gland are often optional, and the sensitivity to radiotherapy of the pituitary gland, which differs between hormonal axes, seems to be underestimated in routine practice, as seen by the frequent lack of endocrine monitoring. However, modern techniques are increasingly efficient in generating steep dose gradients that could help to spare the critical organs and PG. Moreover, the technical potential for PG sparing appears higher for non-PG tumors, but other challenges, including brain sparing from intermediate–low doses, are increasingly crucial for both PG and non-PG tumors. More systematic and prospective studies and assessments of the benefits of endocrine replacement are warranted. They may help refine pituitary and hypothalamus dose constraints further to exploit the sparing potential of modern radiotherapy techniques.

## Figures and Tables

**Table 1:** Studies with data on hypopituitarism following irradiation for non-pituitary tumors.

Author	Patients	Age at RT (Years) (Range)	Technique	Diagnosis	Median Follow-Up (Range)	Mean Dose to PG (Gy)	New Pituitary Impairment (%)						Median Time Since RT before Diagnosis of New Deficit (Years) (Range)					
							Any	Prolactin	ACTH	TSH	FSH/LH	GH	Any	Prolactin	ACTH	TSH	FSH/LH	GH
Partoune 2021 [7]	48	49.2 ± 12.0	3DCRT (15); IMRT (33)	Meningioma	7.5 (1.4–18.1)	48.9 (6.0–55.1)	38	11	13	32	28	35	NR	NR	5.0 (1.0–9.2)	1.9 (0.3–4.8)	2.2 (0.9–6.0)	1.9 (0.3–6.5)
Raymond 2021 [8]	52	56.2 ± 14.1	NR	Meningioma	7 (5.0–10.0)	47 ± 9.4	60.2	18.5	15.4	28	36.9	13.4	NR	NR	NR	NR	NR	NR
Gebauer 2020 [9]	26	58 (36–81)	WBRT (moderate hypofractionation)	Prophylactic for SCLC or therapeutic for brain metastases (NSCLC. SCLC. breast or urothelial)	1.7 (0.5–12.6)	34.4 (30–41.25)	50	26.9	4.8	5	37.5	8	NR	NR	NR	NR	NR	NR
Tabrizi 2019 [10] *	20	37.5 (22–56)	Proton	Glioma	6.8	Dmax > 20 Gy RBE (60% half of which > 30 Gy), Dmax < 5 Gy RBE (40%)	30	NR	20	15	10	0	0.9 (0.4–3.15)	NR	NR	NR	NR	NR
Lamba 2019 [11]	74	53 (13–83)	Proton or photon (NR, including 2 SBRT)	Meningioma	3.6 (0.25–20.8)	51.4 (0.6–61.5)	20	15	24	24	10	19	NR	1.0 (0.75–1.08)	2.67 (0.17–8.67)	1.5 (0.75–3.25)	1.0 (0.75–2.5)	0.92 (0.58–2.5)
Kyriakakis 2019 [12]	58	41.2 ± 10.9	3DCRT	Glioma	8.2 ± 5.2	36.7 ± 15.9	84.5	10.3	19	6.9	20.7	82.8	NR	NR	NR	NR	NR	NR
Handisurya 2019 [13] *	436	50 (19–83)	3DCRT	Glioma	NR	NR	NR	38.3	NR	9.2	NR	NR	NR	NR	NR	NR	NR	NR
Kyriakakis 2016 [14]	107	40.0 (26.9–53.1)	3DCRT	Various brain tumors	8	NR	88.8	15	23.4	11.2	34.6	86.9	NR	NR	3.9 (2.5–5.7)	5.3 (1.8–14.2)	4.6 (2.3–7.9)	3.3 (2.1–5.0)
De Marzi 2015 [15]	103	NR	Photon 3DCRT and proton	Chordoma or chondrosarcoma	NR	54.0 (1.8–72.8)	44	29	NR	11	NR	NR	NR	NR	NR	NR	NR	NR
Seland 2015 [16]	140	42.5 (15–76)	NR	Haematological (93%)	16.1 (15–29)	13.0 (0–68.5)	14	NR	NR	1.4	7.9	5	NR	NR	NR	NR	NR	NR
Ipekci 2015 [17]	30	45.2 ± 9.8	3DCRT or Co60	Nasopharyngeal	2.0 (0.8–11.1)	46.23	93.3	43.3	73.3	26.7	6.7	76.7	NR	NR	NR	NR	NR	NR
Appelman-Dijkstra 2014 [18]	80	47.5 (18.6–89.7)	NR	Various brain and nasopharyngeal tumors	6.0 (0.5–35.0)	56.27 (40.0–70.0)	62	21	31	14	25	33	NR	2.5 (0.5–21.0)	6.0 (0.5–24.0)	5.1 (1.5–10.3)	7 (0.5–16.0)	4.5 (0.5–35.0)
Ratnasingam 2015 [19]	50	50 ± 6.7	NR	Nasopharyngeal	8 (3–21)	>40	82	30	40	4	22	78	NR	NR	NR	NR	NR	NR
Sara 2011 [20] *	26	38.5 (33–47)	3DCRT	Various brain tumors	2.67 (1.0–9.25)	41.8 (30.7–49.8)	38	0	22	14	4	29	1.92 (1.75–2.5)	NR	NR	NR	NR	NR
Minniti 2011 [21] *	52	56 (34–74)	SBRT	Meningioma	3.5 (0.75–6.0)	NR	19	NR	7.7	7.7	13.5	17.3	3	NR	NR	NR	NR	NR
Snyers 2009 [22]	21	61(27–74)	3DCRT	Sinonasal	8.9 (0.9–21.1)	51–56	62	9.5	19	14.3	19	23.8	NR	NR	NR	NR	NR	NR
Bhandare 2008 [23]	312	NR	3DCRT	Sinonasal	5.6	62.4 (39–73)	14.1	3.2	4.5	5.4	3.8	5.1	5.6 (4.5–8.1)	NR	NR	NR	NR	NR
Schneider 2006 [24]	44	NR (20–79)	NR	Various brain tumors	NR	NR	38.6	6.8	18.2	15.9	29.5	27.3	NR	NR	NR	NR	NR	NR
Agha 2005 [25]	56	33.3 (21.3–45.3)	NR	Various brain tumors	3.2 (1.0–12.5)	BED 54 (4–97)	41	32	21	9	27	32	NR	NR	NR	NR	NR	NR
Johannesen 2003 [26]	25	38 (14–68)	3DCRT	Glioma	13.1 (6.0–25.6)	54.0 (45.0–59.4)	64	NR	4	56	28.6	NR	NR	NR	2	5.0 (0.3–15.0)	0.1 (0.1–3.0)	NR
Pai 2001 [27]	107	41.2 (17–75)	Proton and photon	Chordoma or chondrosarcoma	5.5	<68.4 (55.8–79)	87	72	19	30	29	NR	NR	2.5 (0.6–14.3)	3.6 (1.0–14.3)	3.6 (0.5–14.3)	4.0 (2.2–11.7)	NR

Legend: NR: not reported; *: a prospective study.

**Table 2:** Studies with data on hypopituitarism following irradiation for pituitary adenomas.

Author	Patients	Age at RT (Years) (Range)	Technique	Median Follow-Up (Range)	Tumor Volume (cm^3^)	Mean Dose to PG (Gy)	New Pituitary Impairment (%)					Median time Since RT before Diagnosis of New Deficit (Years) (Range)				
							Any	ACTH	TSH	FSH/LH	GH	Any	ACTH	TSH	FSH/LH	GH
Sumodhee 2022 [28]	29	54 (23–86)	SBRT	3.9 (1.0–10.1)	4.8 (0.4–14.7)	35	17	3.4	3.4	NR	NR	NR	NR	NR	NR	NR
Graffeo 2021 [29]	97	50 (38–57)	SRS	4 (2.8–5.7)	2.8 (1.4–4.4)	15 (non-secreting)/25 (secreting)	28	16	14	14	4	1.8 (1–3)	NR	NR	NR	NR
Uygur 2020 [30]	110	49 ± 12	SRS	6.5 ± 4.7	NR	23.3 (16–30)	5.4	0.9	0.9	2.72	NR	NR	NR	NR	NR	NR
Zibar Tomšić 2017 [31]	27	49 (23–74)	SRS	6 (0.5–12)	3.4 (0.1–16.8)	14.4 (5.9–22.8)	30	19	8	14	NR	3.5 (0.25–8.0)	NR	NR	NR	NR
Cohen-Inbar 2016 [32]	60	41.5 (18–69)	SRS	13.3 (5.0–23.2)	1.3 (0.3–13.4)	25 (6–30)	58.3	18.3	26.7	28.3	33.3	5.1 (1.0–13.3)	>10	5 < x < 10	5 < x < 10	5 < x < 10
Iwata 2016 [33]	52	35 (14–67)	SBRT	5 (2.3–11.4)	4.4 (0.2–19.8)	21	1.9	NR	NR	NR	NR	NR	NR	NR	NR	NR
Puataweepong 2016 [34]	40	49.5 (26–68)	SBRT	3.2 (1.2–5.9)	3.4 (0.8–25.9)	25 (20–28)	0	0	0	0	0	NR	NR	NR	NR	NR
Boström 2015 [35] *	35	54 (30–75)	SRS/normofractionated SBRT	8 (2–13)	1.2 (SRS)/8.0(SBRT)	20 (SRS)/54 (SBRT)	NR	46.4	NR	NR	NR	NR	NR	NR	NR	NR
Lee 2014 [36]	136	44 (14–93)	SRS	5.1	2.3 (0.3–16)	25 (8.8–30)	31.6	NR	NR	NR	NR	4.2 (0.7–10.6)	NR	NR	NR	NR
Starke 2012 [37]	140	51 (21–82)	SRS	4.2 (0.5–17)	5.6 ± 5.6	18 (6–25)	30.3	13.8	28.1	7.6	11.8	NR	NR	NR	NR	NR
Park 2011 [38]	88	53.7 (15.5–88.1)	SRS	5.3 (0.5–15.1)	3.5(0.4–28.1)	13	24	10.7	9.7	6.9	5.7	2 (0.25–9.5)	NR	NR	NR	NR
Iwata 2011 [39]	74	59 (16–82)	SBRT	2.8 (1.8–9.9)	5.1 (0.7–64.3)	21/25	4.1	NR	NR	NR	NR	NR	NR	NR	NR	NR
González 2011 [40]	40	52.9 ± 12.1	3DCRT	8 (7–12)	1.9	52	NR	15	7	5	NR	NR	NR	NR	NR	NR
Sheehan 2011 [41]	418	44 (12–91)	SRS	2.6 (0.5–10.3)	1.9 (0.1–27)	24 (9–30)	24.4	NR	NR	NR	NR	NR	NR	NR	NRN	NR
Feigl 2010 [42]	108	51.9 (9.1–81.2)	SRS	6.7	6 (0.2–80)	11.0 (2.3–31.1)	43.5	17.2	25.3	27.3	24.2	NR	3.0 (0.25–9.6)	2.33 (0.25–9.3)	2.25 (0.25–9.3)	2.6 (0.25–4.7)
Leenstra 2010 [43]	82	48	SRS	5.3 (1.1–11.2)	2.7 ± 2.7	20 (11–30)	41	14	22	21	13	2.67 (0.2–9.8)	2.25 (1.0–9.2)	2.25 (0.2–4.7)	2.3 (0.4–6.7)	2.7 (1.1–9.8)
Castinetti 2009 [44]	76	42.7 (7–66)	SRS	8 (5–13)	0.5 (0.2–0.9)	25 (12–40)	22.4	11.8	9.2	14.5	6.6	4 (1–6)	NR	NR	NR	NR
Langsenlehner 2007 [45]	87	51.3 (22.7–79.8)	3DCRT	10.5 (1.4–20.8)	NR	50.4 (46–54)	88	59	64	96	98	NR	7.08 (5.61–11.9)	4.67 (3.1–6.91)	1.96 (1.5–3.53)	NR
Liščák 2007 [46]	79	54 (24–73)	SRS	5 (3–10)	3.5 (0.1–31.3)	20	2.5	1.3	1.3	0	0	NR	4	7	NR	NR
Mingione 2006 [47]	61	51.1 (21–82)	SRS	4 (0.5–10.6)	4.8 (0.6–27)	18.5 (5–25)	19.7	6.6	14.8	NR	3.3	2.2 (0.7–8.9)	1.4 (0.9–2.1)	2.3 (0.7–8.9)	NR	2.2 (1.1–3)
Jenkins 2006 [48]	656	48 (14–80)	3DCRT	7 (3–13)	NR	45 (10–55)	NR	15	27	18	NR	NR	NR	NR	NR	NR
Pouratian 2006 [49]	28	43.1 (17–71)	SRS	4.3 (1.25–10.2)	3.0 (0.2–10.6)	18.6 (0.3–25)	29	7	18	NR	7	3.7 (2.75–4.25)	NR	NR	NR	NR
Colin 2005 [50] *	110	50 (6–83)	Normofractionated SBRT	6.7 (4.0–13.1)	NR	50.4	36.7	28.6	32.3	13.9	NR	NR	NR	NR	NR	NR
Pollock 2002 [51]	43	42	SRS	3 (1–9)	4.3 (0.4–17.3)	20 (14.4–30)	16	12	14	5	NR	5	NR	NR	NR	NR

Legend: SBRT is hypofractionated if not otherwise specified. NR: not reported; *: a prospective study.

**Table 3:** Pros and cons of radiotherapy techniques.

	Pituitary Tumors	Non-Pituitary Head and Neck, Sinonasal and CNS Tumors
	Total dose on PG > 45 Gy (dose per fraction); No advantage on PG, any axis, between techniques	Wide range of total doses (examples: (sinonasal carcinoma = 70 Gy, meningioma 50–54 Gy) on PG may be <20 to 60 or over
Conformal radiotherapy (head and neck and CNS irradiations before the year 2000, WBRT, TBI)	(2–4 Gy/f) No optimization on PG nor a healthy brain around tumor	(2–4 Gy/f), if using two opposed beams leaves little potential for dose optimization on PG (and might not be considered a priority versus other structures) In WBRT, dose to the brain = dose to the PG
Intensity-modulated radiotherapy	(≈1.8–2.2 Gy/f) Steep dose gradients, low dose bath ≈2–5 Gy to whole brain	Standard in most brain and head and neck primary tumors (≈<<2 Gy/f for tumors > 2 cm from PG) For tumors distant > 2 cm from PG, PG dose optimization might be reduced further. If PG is in the low-risk target volume (50 Gy in head and neck tumors), one might need to evaluate the trade-off, i.e., low-risk target volume coverage versus lower dose to PG, if decreasing the PG dose using extreme dose modulation improves the quality of life without increasing tumor failure risks
Stereotactic body radiotherapy	Frequently used for PG adenomas (≈4–5 Gy/f, sometimes ≈2 Gy/f) Very steep gradients ≈2 Gy to the whole brain Yet, significant dose to the carotids and nerves in the cavernous sinuses: Long-term vascular and neurotropic effects?	Frequently used for brain metastases (≈4–5 Gy/f) For tumors within 1 cm from PG, the PG dose may be optimized For tumors > 1 cm from PG, the PG dose could be lower than 20 Gy in most situations Dose to brain and radionecrosis might remain clinical issues in the mid-long term
Proton therapy	(≈2 Gy/f, in 2022 but arctherapy, hypofractionation and flash proton therapy may change this regimen), steep dose gradients ≈5–10 Gy in the 2–3 proton beams, no dose in the brain elsewhere	(≈2 Gy/f) For tumors within 1 cm from PG, PG dose may be optimized using technical sophistication (such as better range shifters, multi-field optimization, proton overshoot of in-front beams, DECT and reduced robustness constraints etc.) but not necessarily better than photon techniques. However, the proton beam allows significant sparing of distant brain tissue. For tumors > 1 cm, PT might be superior to IMRT and SBRT by avoiding low doses to the brain and vessels

## Data Availability

Not applicable.
